# Gliomas and epilepsy: Insights from neuropathological studies in humans

**DOI:** 10.1186/2193-1801-4-S1-L9

**Published:** 2015-06-12

**Authors:** Eleonora Aronica

**Affiliations:** Department of (Neuro)Pathology, Academic Medical Center and Swammerdam Institute for Life Sciences, Center for Neuroscience University of Amsterdam, The Netherlands, SEIN – Stichting Epilepsie Instellingen Nederland, Heemstede, The Netherlands

**Keywords:** Glioma, glioneuronal tumor, epilepsy

Brain tumors represent a recognized cause of epilepsy in both children and adults. In principle, any tumor (extra-axial, intra-axial, benign or malignant, common or uncommon) can cause seizures. However, patients with supratentorial low-grade glial tumors are more likely to develop epilepsy. Several clinical studies emphasize that pharmacologically intractable epilepsy critically affects the daily life of patients with brain tumors, even if the tumor is under control. Recently, the term of long-term epilepsy associated tumour (LEAT) has been introduced. LEATs are low grade, slowly growing, cortically-based tumours which predominantly occur in young patients with long histories (often 2 years or more) of drug-resistant epilepsy. Glioneuronal tumors (GNT), including gangliogliomas (GG) and dysembryoplastic neuroepithelial tumors (DNTs), represent the most common tumor within the spectrum of LEAT. The advent of the neurosurgical treatment of epilepsy-associated brain lesions confirmed the strong epileptogenicity of these tumor entities. Understanding the mechanisms that underlie epileptogenesis in LEATs is essential to identify new treatment targets and to develop an effective therapy. Mechanisms such as alterations of the balance between excitation and inhibition and alterations in neuron-glia interactions might be involved. Astroglial cells express functional receptors for a variety of neurotransmitters and may critically modulate synaptic transmission. In addition, an increasing number of observations indicate that pro-epileptogenic inflammatory pathways are activated in GNT and may contribute to the onset and progression of seizures. The recent advances and likely candidate mechanisms and molecules involved in tumor-associated epileptogenesis will be discussed.

